# Scent of Jasmine Attracts Alien Invaders and Records on Citizen Science Platforms: Multiple Introductions of the Invasive Lacebug *Corythauma ayyari* (Drake, 1933) (Heteroptera: Tingidae) in Italy and the Mediterranean Basin

**DOI:** 10.3390/insects11090620

**Published:** 2020-09-10

**Authors:** Giuseppe Mazza, Luca Nerva, Agostino Strangi, Emiliano Mori, Walter Chitarra, Attilio Carapezza, Maurizio Mei, Leonardo Marianelli, Pio F. Roversi, Alessandro Campanaro, Fabio Cianferoni

**Affiliations:** 1 Research Centre for Plant Protection and Certification, Council for Agricultural Research and Economics (CREA-DC), Via di Lanciola 12/a, Cascine del Riccio, 50125 Firenze, Italy; giuseppe.mazza@crea.gov.it (G.M.); agostino.strangi@crea.gov.it (A.S.); leonardo.marianelli@crea.gov.it (L.M.); piofederico.roversi@crea.gov.it (P.F.R.); alessandro.campanaro@crea.gov.it (A.C.); 2 Institute for Sustainable Plant Protection, National Research Council of Italy (CNR-IPSP), Strada delle Cacce 73, 10125 Torino, Italy; luca.nerva@crea.gov.it (L.N.); walter.chitarra@crea.gov.it (W.C.); 3 Research Centre for Viticulture and Enology, Council for Agricultural Research and Economics (CREA-VE), Via XXVIII Aprile 26, 31015 Conegliano (Treviso), Italy; 4 Institute of Research on Terrestrial Ecosystems, National Research Council of Italy (CNR-IRET), Via Madonna del Piano 10, 50019 Sesto Fiorentino (Firenze), Italy; emiliano.mori@cnr.it; 5 University of Palermo, Via Sandro Botticelli 15, 90144 Palermo, Italy; attilio.carapezza@unipa.it; 6 Entomology, Department of Biology and Biotechnology “Charles Darwin”, Sapienza University of Rome, Piazzale Valerio Massimo 6, 00142 Roma, Italy; maurizio.mei@uniroma1.it; 7 Zoology, “La Specola”, Natural History Museum, University of Florence, Via Romana 17, 50125 Firenze, Italy

**Keywords:** alien invasive species, citizen science, occurrence, *Jasminum*, lacebug, ornamental plants, tingid

## Abstract

**Simple Summary:**

The distribution of the lacebug *Corythauma ayyari*, a pest species associated to jasmine plants, has been updated using collections and citizen-science data. The path of introduction of this species in Italy has been inferred with molecular analysis. The results revealed an extent of occurrence in Italy wider than was previously known and the evidence of multiple introduction events. The work shows that citizen science can represent a further tool within the early warning information system for alien species introduction.

**Abstract:**

The jasmine lacebug *Corythauma ayyari* is a pest of cultivated and ornamental plants mainly associated to *Jasminum* spp. This invasive insect is native to Asia, and it has been recently introduced in several countries, mainly within the Mediterranean basin. Here, we updated the known distribution of this species, including five new Italian regions (Liguria, Tuscany, Latium, Apulia, and Calabria); Salamis Island in Greece, and the Occitanie region in France. Citizen-science data have significantly contributed to the knowledge on species distribution, and the online platform for sharing biodiversity information can represent an effective tool for the early detection. Molecular analyses revealed that the specimens collected in Peninsular Italy and Sicily belong to a unique clade, suggesting the possibility of a single introduction, whereas those from Menton (France) and Calabria (Southern Italy) are separated from the others and probably originate from separated introductions.

## 1. Introduction

In the last three decades, the number of animal-watchers reporting photos and observations of animal species on websites, citizen-science platforms, forums, and social networks has exponentially increased [1,2,3,4,5,6,7]. This is particularly evident for alien invasive species in the surroundings of human settlements [8] or damaging cultivated and ornamental plants [9,10,11].

Among those, the jasmine lacebug *Corythauma ayyari* (Drake, 1933) is an invasive insect (Hemiptera: Heteroptera: Tingidae) native to Southern Asia from Pakistan to Indonesia (distribution summarized by [12]). In the last 20 years, the species has been recorded from Israel in 2004 [13], the United Arab Emirates in 2005 [14], France in 2009 [15], Italy in 2012 [16], Tunisia in 2013 [17], Malta [18] and Spain in 2014 [19], Greece in 2015 [20], Syria [21] and Egypt in 2017 [22], and the Principality of Monaco in 2019 [23].

As in Italy, the jasmine lacebug was detected for the first time in 2012 in Campania (Southern Italy) on common jasmine *Jasminum officinale* L. [16] and then, in 2014, on Spanish jasmine *J. grandiflorum* L. in Sicily by [12]; in 2013 and 2019, the alien insect was found also in Sardinia [24].

*Corythauma ayyari* is a quite peculiar species showing an elongate-oval body about 2.5–3.0 mm long, characterized by the presence of a subspherical pronotal hood strongly elevated above medial carina. These morphological characters allow us to distinguish the species from other tingids occurring in Mediterranean countries [14] and are, therefore, easily identifiable also by non-experts, which makes the species a good candidate for a citizen-science campaign.

This lacebug has been reported on a variety of plant species belonging to different families, and it is regarded as a serious pest for cultivated and ornamental plants, e.g., *Eranthemum pulchellum* Andrews (Acanthaceae), *Trachelospermum* sp. (Apocynaceae), *Ocimum* sp. and *Volkameria inermis* L. (Lamiaceae), *Althaea officinalis* L. (Malvaceae), *Musa* sp. (Musaceae), several species of *Jasminum* (Oleaceae) [*J. azoricum* L., *J. grandiflorum* L., *J. multiflorum* (Burm.f.) Andrews, *J. officinale* L., *J. sambac* (L.) Aiton], *Lantana* sp. (Verbenaceae), and *Hedychium* sp. (Zingiberaceae) [13,15,16,17,19,22,25]. Both adults and nymphs feed on sap from leaves of host plants; infected leaves show chlorosis, and eventually, they desiccate [16,17,18,19].

General insecticides and the destruction of infected leaves has been recommended to control this pest [14], but because of its limited known occurrence and of the low number of affected host plants, no study to manage it has been performed yet.

Aims of this article are (1) to update the known distribution of this species with a focus on Italy; (2) to evaluate if citizen science has represented a useful approach towards an early warning and information system for alien species; (3) to assess the potential origin of the Italian populations by means of genetic analyses.

## 2. Material and Methods

We updated the distribution of *C. ayyari* by searching for occurrences in the scientific literature, citizen-science platforms, social networks, and photo/video-sharing websites. Redefinition of its range was plotted on a map. Further unpublished data were obtained from occasional samplings by some of the authors.

Samples of *C. ayyari* were directly collected on infested plants and preserved in absolute ethanol until the morphological and molecular identifications. Some specimens were dry-mounted for the morphological study. All collected samples were morphologically analyzed under a stereomicroscope Nikon SMZ-1500 (Nikon Corporation, Tokyo, Japan) equipped with a cold light source.

Further data on this species concerning distribution and host plants come from citizen science: The Italian naturalistic forum “Forum Entomologi Italiani” (www.entomologiitaliani.net) and “iNaturalist”, the system for sharing biodiversity records (www.inaturalist.org). Published articles based on citizen-science records were also analyzed. Concerning occurrences all the available sources, social networks (Facebook, Twitter, and Instagram), searching for the species on entomological groups, as well as websites of photo and video-sharing (i.e., Flickr and YouTube), and other naturalistic sites, were checked. *Corythauma ayyari* and “jasmine lacebug” were used as keywords for searching online, and the last check of the data was made in July 2020. All the citizen-science records were validated by examination of the available picture(s). The plants’ nomenclature follows the online source “The Plant List” (http://www.theplantlist.org/).

For each site, the following information are given as: region, locality, coordinates, date, collector or photographer, number of specimens, sex and life cycle stage (when available), possible repository. Records are listed geographically from west to east, and Italian regions are arranged from north to south.

Geographical coordinates are in decimal degrees, with datum WGS84. Number of decimals is proportional to the accuracy; uncertainty was indicated as it appears on each iNaturalist observation.

Regarding the specimens collected, if the repository is not specified, the entire individual was used for molecular analyses (m.a.); remaining specimens after molecular analysis were preserved as voucher specimens (CFC = collection F. Cianferoni, Florence, Italy).

Total DNAs were extracted from individual adult specimens using DNeasy Blood & Tissue DNA extraction kit (Qiagen, Venlo, Netherlands), according to the manufacturer’s protocol. Amplification of the barcode region of *COX I* gene (which hosts the COI region) was obtained as described in [26], this locus was chosen to reconstruct the molecular phylogenies of samples collecting related sequences in the Barcode of Life Data System (BOLD) and National Center for Biotechnology Information (NCBI) databases [27].

The *CytB* mitochondrial gene was further amplified and sequenced to build a phylogenetic tree [28]. The obtained sequences (*COX* and *CytB*) were concatenated in a single long sequence; the same data were retrieved for the reference sequences retrieved from the NCBI database: *Corythucha ciliata* (Say, 1832) and *Cimex lectularius* Linnaeus, 1758. Sequences used for inferring the tree are reported in Table S1. Pairwise alignments of concatenated sequences were performed using Muscle [29]. The best fitting evolutionary model for further phylogenetic analysis was selected using the algorithm implemented in MEGA7 [30]. The Tamura model with Gamma distribution (T92 + G8) resulted in the best model for the considered data. Evolutionary relationships were inferred with MEGA7 [30], using the maximum likelihood (1000 bootstrap replicates) methods based on the Tamura–Nei model [31], as previously reported [32].

## 3. Results

**Figure 1:**
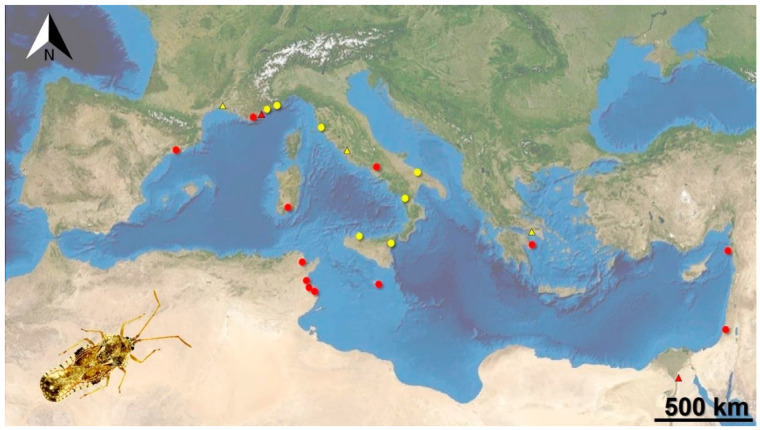
Map of known occurrences of the alien lacebug *Corythauma ayyari* in the Mediterranean basin. Dots represent collected specimens and triangles, indicating records from citizen science; the published occurrence records are in red, and the new data from our work are in yellow (US Dept of State Geographer, © 2020 Google, Map Data © 2020 AND Image Landsat/Copernicus).

The jasmine lacebug *Corythauma ayyari* currently occurs in seven Italian regions, also including five new ones for which no records were known yet (Figure 1 and Table S2). We directly collected samples from all of them but not for Campania and Sardinia.

Material examined (collected specimens): FRANCE. Provence–Alpes–Côte d’Azur: Menton, 43.774156° N, 7.490061° E, 14 m a.s.l. on cultivated *Jasminum officinale*, 14.VIII.2018, seven specimens (m.a). ITALY: Liguria. Santo Stefano al Mare (Imperia), Creuza de Ma farmhouse, 43.84027° N, 7.90502° E, 108 m a.s.l., on cultivated *Jasminum officinale*, 11.VIII.2018, G. Mazza & E. Mori leg., three specimens (m.a.). Tuscany. Piombino (Livorno), Torre Mozza, 42.94545° N, 10.69779° E, 1 m a.s.l., on cultivated *Jasminum officinale*, 20.VIII.2018, E. Mori leg., two specimens (m.a.). Latium. Roma, Tor Sapienza, 41.903750° N, 12.586602° E, on potted pink-flowers *Jasminum* sp., 31.VIII.2016, M. Mei leg., 18 males, 15 females, 1 V, 1 IV, 2 III instar nymphs (CFC). Apulia. Bari, 41.12807° N, 16.87150° E, 4 m a.s.l., on potted *Jasminum officinale*, 29.IX.2016, G. Mazza & L. Marianelli leg., two specimens (m.a.). Calabria. San Nicola Arcella (Cosenza), 39.848676° N, 15.794861° E, 124 m a.s.l., on potted *Jasminum officinale*, 13.VIII.2016, G. Mazza & E. Mori leg., 11 males, 10 females (CFC). Sicily. Castelmola (Messina), Castello, 37.8590° N, 15.2775° E, 530 m a.s.l., on potted *Jasminum officinale*, 12.VIII.2015, G. Mazza & E. Tricarico leg., 2 males, 5 females (CFC); Palermo, 38.1394° N, 13.3360 E, 70 m a.s.l., on potted *Jasminum grandiflorum*, 14.IX.2016, A. Carapezza leg., 11 males, 9 females, 11 V, 5 IV instar nymphs (CFC).

Several occurrence data were retrieved from citizen-science sources: the iNaturalist platform and the naturalistic forum “Forum Entomologi Italiani”. All records were listed below, including published records, which were also examined.

Material examined (citizen-science data): FRANCE. Occitanie: Montpellier, 43.610769° N 3.876716° E (uncertainty = 7.03 km), 18.X.2019, one specimen, photo by “jtch” (www.inaturalist.org; id 34558936). MONACO. 43.735323° N 7.414166° E, on *Jasminum azoricum*, 30.VIII.2019, several specimens, photo by Robin Duborget (www.inaturalist.org; id 32574772) [22]. ITALY. Latium, Roma, Tor Sapienza, 41.903750° N 12.586602° E, on potted pink flowers *Jasminum* sp., 26.X.2014, many specimens, photos by Maurizio Mei (www.entomologiitaliani.net). GREECE. Salamis Island, 37.929079° N 23.513327° E (uncertainty = 10.07 km), 20.V.2014, one specimen, photo by Vaggelis Koutsoukos (www.inaturalist.org; id 14981293). EGYPT. Cairo, Al Zaitoun, 30.10109° N 31.297109° E (uncertainty = 10 m), 1.VI.2017, one specimen, on *J. sambac,* photo by Yasmin Abdel Monem (www.inaturalist.org; id 6467128) [23].

No other occurrence was retrieved from social networks and photo/video-sharing websites.

Specific primers successfully amplified a ~680 bp DNA fragment for the *COX* gene and a ~820 bp DNA fragment for the *CytB* gene. A database search for amplified sequences gave different results: for the *COX* gene high similarities were found in Tingidae sp. samples from Pakistan (100% identity with KY839550.1 and 99.79% identity with KY841149.1), the native area of *C. ayyari*. In contrast, search for *CytB* sequences displayed only some low similarity (80% identity) with *Perissonemia borneensi* (Distant, 1909). Due to absence of both *CytB* and *COX* sequences for *Corythauma* species, we deposited the amplified sequence in NCBI under accession: MT476316–MT476327 and MT478933–MT478944 for *CytB* and *COX*, respectively.

**Figure 2:**
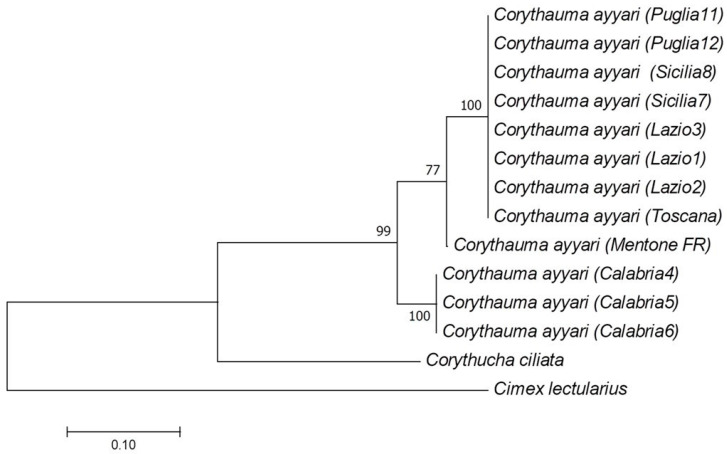
Phylogenetic tree derived from the pairwise alignment of *COX* and *CytB* sequences amplified from selected specimens and from two NCBI retrieved samples. The percentage of trees in which the associated taxa clustered together is shown next to the branches. The tree is drawn to scale, with branch lengths measured in the number of substitutions per site.

Phylogenetic analysis of the amplified sequences (Figure 2) revealed the presence of at least two subgroups in the *Corythauma* specimens analyzed: the first is formed by individuals coming from Apulia, Tuscany, Latium, Sicily, and France. The second well-supported clade (99% bootstrap confidence) is formed by all the specimens from Calabria.

## 4. Discussion

Our work reviewed the distribution of the invasive *C. ayyari* in Europe and in the Mediterranean basin. Particularly, before this work, in Italy, this species had been only recorded for Campania, Sicily, and Sardinia [12,16,24]. New data collected in this work provided evidence that the extent of occurrence in Italy is remarkably wider than previously believed.

Currently, based on available data, this pest occurs in some Tyrrhenian regions and several sites of Southern Italy; to date, it is almost completely restricted to Peninsular Italy and major islands and lacking in most of Northern Italy (except for Liguria) and most of the Adriatic regions (with the exception of Apulia). However, further field research and monitoring would be necessary to assess the real distribution of this alien invasive species in Italy.

A further record detected in May 2014 in Greece (Salamis Island) from citizen science (online platform) is also reported, which anticipates the record of June 2015 for the island of Poros [20]. The introduction of this pest in Greece, which so far appeared as limited to the Attica region, was already present at least one year earlier than reported [20].

An additional recent record from citizen science in the Occitanie region (Montpellier, 2019) confirmed the occurrence of this lacebug in Southern France (Mediterranean), where it was already known from the Provence-Alpes-Côte d’Azur region ([15], confirmed by our record from Menton in 2018). Outside its native range, *C. ayyari* is currently restricted to the Mediterranean basin, probably as limited by temperature. Accordingly, South Eastern Asian pests are widespread in warm regions, where introduced [33]. An updated monitoring of alien insect distribution is therefore crucial, as the current global warming may bring them to invade new areas where previously absent [34].

The record from the United Arab Emirates [14] suggests that a possible introduction is ongoing also in other countries of the Middle East (e.g., the Arabian Peninsula).

Citizen-science data significantly expanded the known area colonized by this species, which was previously known (cf. [22,23] and part of these new records). Moreover, the citizen-science platforms represent an effective tool for the early detection of pest species: first records of the presence in Greece (present contribution), Egypt ([23], first record for Africa), and Monaco [22] come from observations made by citizens.

Jasmine species are tropical plants, very widespread as ornamental plants in private gardens throughout the world and particularly appreciated for their scent and rapid growth. Therefore, it is likely that local infestations with parasites would elicit owners’ attention, thus stimulating curiosity, interest, and in turn, the use of online platforms to report data. This is particularly important in a period of economic crisis [35], which may limit the possibility for a targeted monitoring, also given that the biology of *C. ayyari* is still mostly unknown. Ecological information on *C. ayyari* are scant in the scientific and grey literature [36], with most published works dealing with records of this species in new areas. Research efforts should be aimed at assessing life cycle and actual impact of this species on ornamental plants, as well as on biological control methods. Concerning host plants, specimens collected for this work were all found on *Jasminum* spp., confirming the predilection of this pest for this genus of ornamental plants. The most common Chinese star jasmine *Trachelospermum jasminoides* (Lindl.) Lem. (Apocynaceae) close to the infested plant of *J. officinale* in Calabria was pest-free. Moreover, all the specimens of *T. jasminoides* observed in several occasions in Tuscany, Marche, Veneto, Latium, and Piedmont by G. Mazza, E. Mori, and F. Cianferoni were free of the pest.

Molecular analysis showed that the specimens collected in Tuscany, Latium, Apulia, and Sicily belong to a single clade, suggesting the possibility of a single introduction followed by the spread of this insect over the Italian territories. The French sample, which belongs to the same clade but presents some differences in the nucleotide sequence, is probably related to a separate introduction. Surprisingly, the specimens collected in Calabria are all separated from the others, suggesting the introduction of a different population or at least on different occasions.

In addition, we have also to mention that, the presence of endosymbiotic bacterial species (e.g., *Wolbachia*) could have an effect on silent mitochondrial polymorphism, increasing its rate if compared to symbiont free insects [37]. In this respect, the possibility that Calabria samples host this kind of symbionts has to be taken into consideration, particularly in light of the significant difference observed between these sample and the others *C. ayyari* samples. Furthermore, we also have to take in consideration the possibility that, the limited number of individuals analyzed, the lack of other sequences belonging to *C. ayyari* or at least to other *Corythauma* species, and the limited spatial distribution of samples analyzed (if considering also the native geographical distribution) could have impaired the genetic analysis [38]. The latter hypothesis, together with the conserved morphological traits, seems to suggest a much wider intraspecies phylogenetic distance, which have to be evaluated by further analyses as soon as more sequences will be available in databases.

Taken together, these results highlight the multiple origins of *C. ayyari* species in Italy. Translocations as contaminants with ornamental plants, as described in [39], are considered the most probable pathways for this alien Heteroptera and should be more efficiently regulated and restricted by intensified quarantine inspections of imported plant material from outside and within Europe.

## 5. Conclusions

The present study provided first records of the presence of *Corythauma ayyari* in five Italian regions (Liguria, Tuscany, Latium, Apulia, and Calabria) contributing significantly to update our knowledge about occurrence of this species in the Mediterranean basin. Moreover, our work confirmed that citizen science provides important information to picture the distribution of species and represents an efficient tool for early detection of pest species.

Molecular analysis showed that the specimens spread in the Tyrrhenian regions of Italy, Apulia, and Sicily probably derive from a single introduction, while specimens from Calabria seem to originate from a different introduction event, as well as specimens from southern France, which are near the first Italian clade but probably also related to a separate introduction.

## Supplementary Materials

The following are available online at https://www.mdpi.com/2075-4450/11/9/620/s1. Table S1: Accession numbers of sequences used to infer the phylogenetic distances. Table S2: Occurrence of the alien tingid *Corythauma ayyari* in the Mediterranean basin; the year of first observation and coordinates (decimal degrees; WGS84) are indicated. Records from citizen science are underlined.
