# Supplementary material for: Scent of Jasmine Attracts Alien Invaders and Records on Citizen Science Platforms: Multiple Introductions of the Invasive Lacebug Corythauma ayyari (Drake, 1933) (Heteroptera: Tingidae) in Italy and the Mediterranean Basin
Source: Insects. 2020 Sep 10;11(9):620. doi: 10.3390/insects11090620 (PMC7565189; doi:10.3390/insects11090620)
Supplement: Supplementary file 1 [file insects-11-00620-s001.pdf]

Table S1. Accession numbers of sequences used to infer the phylogenetic distances.

Sample Sequence	*COX* Accession	*CytB* Accession
Cimex lectularius	KJ937979.1	NC_030043.1
Corythucha ciliata	KM021816.1	NC_022922.1
Corythauma ayyari (Menton FR)	MT478944.1	MT476326
Corythauma ayyari (Toscana)	MT478943.1	MT476327
Corythauma ayyari (Lazio-1)	MT478933.1	MT476316
Corythauma ayyari (Lazio-2)	MT478934.1	MT476317
Corythauma ayyari (Lazio-3)	MT478935.1	MT476318
Corythauma ayyari (Calabria 4)	MT478936.1	MT476319
Corythauma ayyari (Calabria 5)	MT478937.1	MT476320
Corythauma ayyari (Calabria 6)	MT478938.1	MT476321
Corythauma ayyari (Sicilia 7)	MT478939.1	MT476322
Corythauma ayyari (Sicilia 8)	MT478940.1	MT476323
Corythauma ayyari (Puglia 11)	MT478941.1	MT476324
Corythauma ayyari (Puglia 12)	MT478942.1	MT476325

Table S2. Occurrence of the alien tingid *Corythauma ayyari* in the Mediterranean basin; the year of first observation and coordinates (decimal degrees; WGS84) are indicated. Records from citizen science are underlined.

Year	Country	Locality	Latitude	Longitude	Uncertainty (m)	Source
2004	Israel	Tel Aviv, Herzliya	32.160707	34.845113	2000	[1]
2009	France	Var, Puget-sur-Argens	43.466958	06.671792	250	[2]
2012	Italy	Campania, Provincia di Caserta	41.217335	14.158087	40,000	[3]
2013	Italy	Sardinia, Cagliari	39.200238	09.147266	750	[4]
2014	Italy	<u>Latium, Rome, Tor Sapienza</u>	41.903750	12.586602	5	present contribution
2014	Greece	<u>Salamis Island</u>	37.929079	23.513327	10,070	present contribution
2014	Spain	Barcelona, Puigmoltó	41.248370	01.767400°	250	[5]
2014	Italy	Sicily, Palermo	38.139395	13.335998	5	[6]
2014	Malta	Verdala Palace (Malaise trap)	35.861408	14.400628	120	[7]
2014	Tunisia	Ariana	36.867780	10.182500	10	[8]
2014	Tunisia	M'Saken	35.731940	10.588060	10	[8]
2014	Tunisia	M'Saken	35.731940	10.588060	10	[8]
2014	Tunisia	Akouda	35.877220	10.565560	10	[8]
2914	Tunisia	Kantaoui	35.893330	10.596940	10	[8]
2015	Italy	Sicily, Castelmola	37.859000	15.277500	5	present contribution
2015	Greece	Poros	37.498613	23.455271	50	[9]
2016	Italy	Latium, Rome, Tor Sapienza	41.903750	12.586602	5	present contribution
2016	Italy	Apulia, Bari	41.128070	16.871500	5	present contribution
2016	Italy	Calabria, San Nicola Arcella	39.848676	15.794861	5	present contribution
2016	Italy	Sicily, Palermo	38.139395	13.335998	5	present contribution
2017	Egypt	<u>Cairo, Al Zaitoun</u>	30.101090	31.297109	10	[10]
2017	Syria	Latakia City	35.523784	35.781735	0	[11]
2018	Italy	Liguria, S. Stefano al Mare	43.840270	7.9050200	5	present contribution
2018	Italy	Tuscany, Torremozza	42.945450	10.697790	5	present contribution
2018	France	Menton	43.774156	07.490061	5	present contribution
2019	France	<u>Montpellier</u>	43.610769	03.876716	7030	present contribution
2019	Monaco	<u>Monaco</u>	43.735323	07.414166	10	[12]
2019	Italy	Sardinia, Cagliari	39.226949	09.101827	1500	[4]

References

1. Novoselsky, T.; Freidberg, A. Note: *Corythauma ayyari* (Drake) (Hemiptera: Heteroptera: Tingidae)—A new pest of ornamentals in Israel. *Phytoparasitica* **2013**, *41*, 149–150.
2. Streito, J.C.; Matocq, A.; Guilbert, E. Découverte d'un foyer de *Corythauma ayyari* (Drake, 1933) et point sur la présence de plusieurs espèces de *Stephanitis* envahissants en France (Hemiptera Tingidae). *L'Entomologiste* **2010**, *66*, 7–12.
3. Pedata, P.A.; Guilbert, É.; Nugnes, F.; Manicini, D. Rinvenimento di una popolazione di *Corythauma ayyari* (Heteroptera Tingidae) su *Jasminum officinale* (Oleaceae): Un nuovo fitofago per l'Italia. *Prot. Delle Colt.* **2013**, *3*, 36–39.
4. Fancello, L.; Cillo, D.; Rattu, A. Prima intercettazione in Sardegna dell'emittero "alieno" *Corythauma ayyari* (Drake, 1933), specie potenzialmente invasiva dannosa a piante ornamentali (Insecta, Hemiptera, Heteroptera, Tingidae). *Mediterr. Nat.* **2019**, *2*, 56–59.
5. Roca-Cusachs, M.; Goula, M. First record of the invasive tingid species *Corythauma ayyari* (Drake, 1933) in the Iberian Peninsula (Insecta: Hemiptera: Heteroptera: Tingidae). *Butll. Inst. Catalana Hist. Nat.* **2014**, *78*, 119–123.
6. Carapezza, A. *Corythauma ayyari* (Drake, 1933) new pest of jasmine in Italy (Heteroptera Tingidae). *Nat. Sicil*. **2014**, *38*, 381–384.
7. Carapezza, A.; Mifsud, D. New records of true bugs (Hemiptera, Heteroptera) from the Maltese Islands. *Bull. Ent. Soc. Malta* **2015**, *7*, 27–50.
8. Haouas, D.; Guilbert, E.; Halima-Kamel, M.B. First report of *Corythauma ayyari* (Drake) (Hemiptera: Tingidae) on Arabian and Spanish jasmine in Tunisia. Bull. *EPPO Bull.* **2015**, *45*, 144–147.
9. Rietschel, S. *Corythauma ayyari* (Drake, 1933)—Erstnachweis der invasiven Tingide in Griechenland (Heteroptera, Tingidae). *Heteropteron* **2015**, *44*, 5–8.
10. Van der Heyden, T. First record of *Corythauma ayyari* (Drake, 1933) (Hemiptera: Heteroptera: Tingidae) in Egypt. *J. Het.Turk*. **2020**, *2*, 1–2.
11. Zeity, M.; Ali, A.Y. First report of the lacebug *Corythauma ayyari* (Drake) (Hemiptera: Tingidae) on *Jasminum grandiflorum* L. and *Jasminum sambac* (L.) from Syria. *EPPO Bull*. **2019**, *49*, 398–400.
12. Van der Heyden, T. First record of *Corythauma ayyari* (Drake) (Heteroptera: Tingidae) in Monaco. *Rev. Chil. Entomol*. **2019**, *45*, 579–581.

© 2020 by the authors. Submitted for possible open access publication under the terms and conditions of the Creative Commons Attribution (CC BY) license (http://creativecommons.org/licenses/by/4.0/).
